# Morphological and molecular characteristics of trematode cercariae isolated from the freshwater snails collected from three paragonimiasis endemic areas in Manipur, India

**DOI:** 10.1007/s12639-025-01883-3

**Published:** 2026-01-31

**Authors:** Umananda Yumnam, Karma G. Dolma, Paron Dekumyoy, Urusa Thaenkham, Kangjam Rekha Devi, Mutum Ranjana Devi, T. Shantikumar Singh

**Affiliations:** 1 Department of Microbiology, Sikkim Manipal Institute of Medical Sciences, Sikkim Manipal University, Tadong Gangtok, Sikkim 737102 India; 2 Department of Helminthology, Faculty of Tropical Medicine, Mahidol University, Bangkok, 10400 Thailand; 3 ICMR-RMRCNE Dibrugarh, Dibrugarh, Assam 786010 India; 4 Institute of Bioresources and sustainable Development (IBSD), Imphal, Manipur 795001 India

**Keywords:** Freshwater snails, Trematode cercariae, Prevalence, Manipur

## Abstract

A study was conducted to identify and characterize trematode cercariae infecting freshwater snails in three paragonimiasis endemic areas namely, Luwangsangbam matai, Motbung, and Moreh of Manipur, India, from 2020 to 2024. A total of 9467 snails belonging to seven species namely *Bortia costula* (2404), *Filopaludina bengalensis* (1869), *Paludomous pustulosa* (1474), *Racesina luteola* (1147), *Melanoid tuberculota* (891), *Radix rufescens* (869) and *Indoplanorbis exustus* (814) were collected and examined for cercarial infection using the shedding and crushing methods. The overall prevalence of trematode infection in snails was 0.26%, with the highest infection rate observed in Moreh (0.41%), followed by Motbung (0.36%) and Luwangsangbam Matai (0.09%). Among the snail species, *Melanoides tuberculata* showed the highest infection rate (0.90%), whereas *Indoplanorbis exustus* and *Paludomous pustulosa* were not infected. Morphological examination revealed nine different morphological types of cercariae: Lophocercous (1), Apharyngeate ocellate furcocercariae (1), Gymnocephalous (2), Xiphidio (1), Furcocercous (2), and Echinostomous (2). Molecular analysis based on 18 S rDNA sequencing and phylogenetic analysis identified six trematode species: *Euryhelmis costaricensis*,* Trichobilharzia stagnicolae*,* Haematoloechus sibiricus*,* Australapatemon burti*,* Echinostoma revolutum*, and *Hypoderaeum conoideum*. Seasonal variations in cercarial infection were observed, with higher rates from late spring to summer and lower rates in winter and early spring. This study provides baseline data on trematode-snail dynamics in the freshwater ecosystems of Manipur, which can be used for future epidemiological research and targeted control strategies.

## Introduction

Freshwater snails belong to the class Gastropoda under the phylum Mollusca and can be found in a variety of aquatic environments (Strong et al. 2008). Several species of freshwater snails act as first intermediate hosts and provide a suitable habitat for the larval stages (rediae and cercariae) of trematodes (Stanicka et al. 2023). Trematodes, commonly known as flukes, are digenetic parasites that require more than one host to complete their life cycle (Wongpim et al. 2023).

The interaction between trematodes and their snail hosts is often specific (Adema and Loker 2024). Some trematode species depend on certain species of snails for their development. Therefore, the geographic distribution of trematode infections is associated with the distribution and abundance of snail hosts. Understanding the geographical distribution of freshwater snails and their role in transmitting trematode infections is critical for the control of parasitic infections (Chai and Jung 2024). The more diverse the snails, the more diverse the trematode species; the more diverse the host, the more diverse the parasite (Duan et al. 2021). Trematode infections, especially those caused by Schistosoma species, are major public health problems in tropical and subtropical regions (Madsen et al. 2022). For example, the spread of *Schistosoma mansoni*,* S. japonicum*, and *S. haematobium* by *Biomphalaria*, *Oncomelania*, and *Bulinus* sp. has resulted in schistosomiasis in endemic areas of Africa and Asia (Hailegebriel et al. 2022; Ponpetch et al. 2021).

Freshwater bodies are abundant in India and provide a suitable environment for the development of snail hosts and trematode parasites (Kamchoo and Chai 2023). Several studies have reported the involvement of freshwater snails in the transmission of trematode infections in various regions of India (Sanil and Janardhan 2019; Tigga et al. 2014; Yumnam et al. 2024). However, no studies on freshwater snail and trematode infections have been conducted until the present study in this part of northeastern India. Situated within the Indo-Burma biodiversity hotspot, Manipur is known for its rich aquatic flora and fauna, including freshwater mollusks and crustaceans (Nahar et al. 2025; Yumnam et al. 2024). The geographical characteristics of the state, coupled with its climate, offer a suitable environment for the coexistence of a wide variety of snails and trematodes (Madsen et al. 2022). Despite their importance in ecosystems, proximity to Assam state of Northeast India, where foodborne and cutaneous trematodiases are common, similar food habits, and agricultural activities in Manipur, trematode infections in freshwater snails in Manipur have been poorly studied. Thus, the public is expose to a great risk of infection of trematodes prevalent in Manipur. Information on the prevalence and diversity of trematodes and freshwater snails in this region will help in understanding the host-parasite relationships, formulating appropriate infection control strategies, and minimizing trematode transmission in humans. This study aimed to identify and characterize freshwater snail host species and trematode species occurring in Manipur, India. Both morphological and molecular studies can be used to identify species. The resultant dataset provides a basis for further epidemiological investigations, planning and implementing health strategies, and conserving biodiversity. In addition, this study will enhance our understanding of the ecological interactions between parasites and their hosts as well as the dynamics of infection transmission.

## Materials and methods

### Study design and sampling technique

**Fig. 1:**
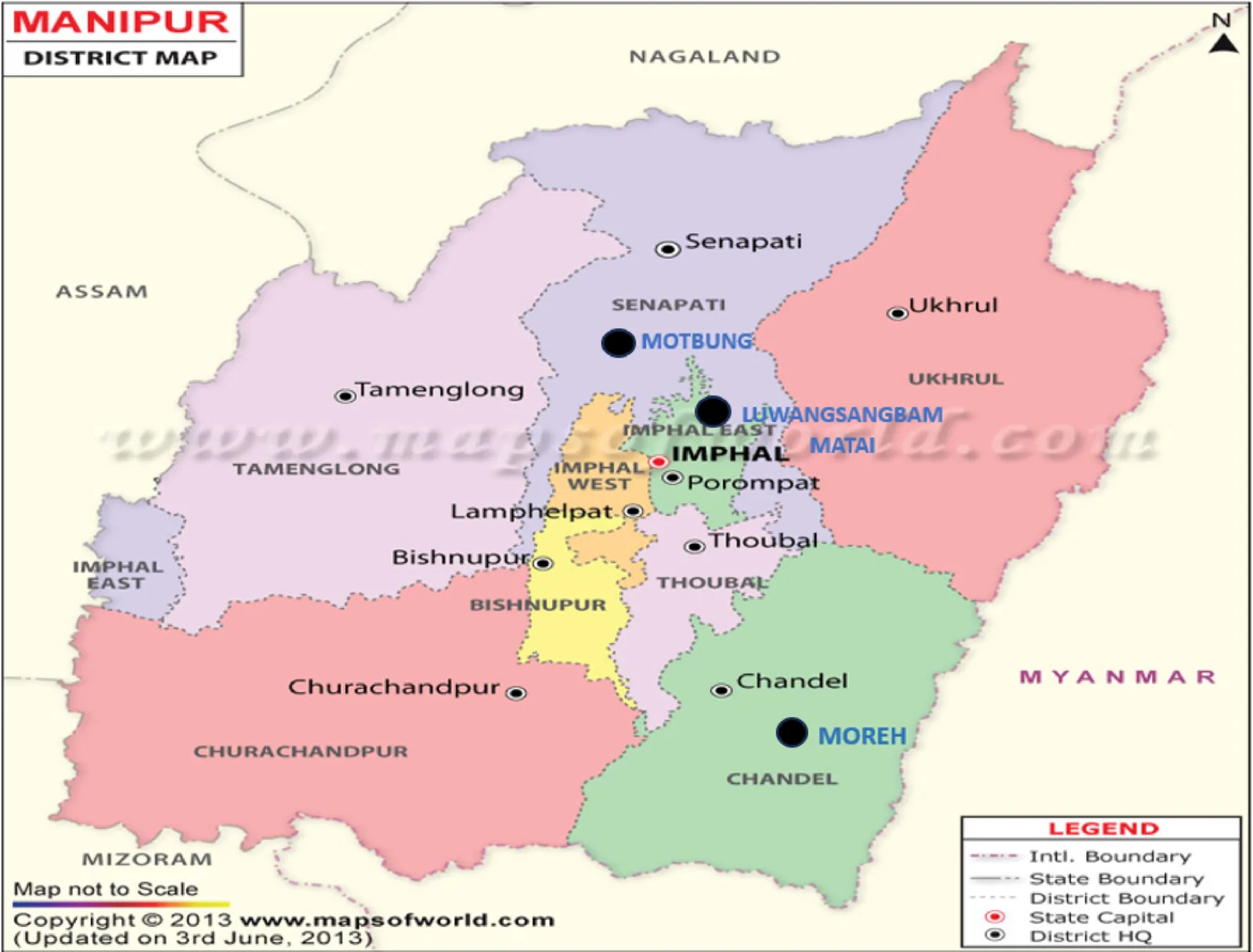
Map of Manipur showing sampling sites for the study

The study was conducted between January 2020 and April 2024 in the known endemic regions of paragonimiasis namely, Luwangsangbam matai in Imphal west, Motbung in Kangpokpi and Moreh in Tengnoupal districts of Manipur, India (Fig. 1). A total of 54 sampling sites, 18 each from three districts were selected and on average, 4–5 visits per collection site were made. Freshwater snails were collected monthly using a purposive sampling technique based on their presence and consent from property owners, where *Paragonimus* metacercariae positive crabs were reported from these areas (Singh et al. 2007, 2012). Snails were collected by handpicking or dragging fishing nets through water at various locations in mountain streams, rivers, paddy fields and dams. Samples were collected between 7 and 8 AM, covering an area of approximately 300 m per site per visit. This time frame is for convenience, increased snail activity and availability, and maintaining time uniformity. After thorough washing and cleaning, snails were transported to laboratory for further examination. In the laboratory, the snails were grouped by species based on their morphological characteristics, such as shell structure, size, colors, and surface texture were the morphological characteristics that were used to classify the snails into species as described by Gupta et al. (1987) and Rao et al. (1989), and further confirmed by experts in the Zoology Department of Manipur University.

### Harvesting and morphological identification of cercariae

Trematode cercariae were harvested from the freshwater snails by using shedding and crushing methods as described by Yaemput et al. (1994). Wet mount and borax carmine-stained smears were used for morphological identification of trematode cercariae. Trematode cercariae isolated from individual hosts were grouped according to their morphological features and species. Ten to fifteen cercariae from each group were preserved in 10% formalin for future reference. The remaining cercariae of each group were preserved in 70% ethanol for molecular analysis.

### Molecular techniques for species identification

Determination of trematode species based on the morphological features of cercariae requires an experienced helminthologist. Difficulties arise because cercariae are small, soft, and possess only a few stable morphological characteristics that are subject to host-induced phenotypic variations (Graczyk 1991). Moreover, these are time- consuming methods and requires a high level of experience-based skills. Hence, molecular identification is an accurate tool for identification of trematode cercariae and also to settle any dispute in the species identification of morphologically similar cercariae in question.

### DNA isolation, amplification and sequencing

DNA samples were prepared from individual cercariae using Nucleospin^®^ Tissue DNA kit (740952.50; Macherey –Nagel Germany) as per manufacturer’s instruction. Final elution of DNA was performed in 100 µL Elution buffer. Quantification of isolated DNA was performed by using 1% (w/v) agarose gel electrophoresis and Qubit estimation. Selective polymerase chain reaction (PCR) enables amplification of the 18 S ribosomal DNA (rDNA) gene, which contains interspecific polymorphic regions (Routtu et al. 2014), using the following primers.

**Table:** 

Primer name	Sequences 5’-3’
B- forward	GCCAAGGATGTTTTCATTGATCT
D- Reverse	TCCTCTAATGATCAAGTTTGGT

Approximately 25ng of genomic DNA and 200nM of each primer i.e. 0.5 µl of each primer having concentration of 10µM was used per reaction for PCR. PCR reaction was performed in Applied Biosystem 2720 thermal cycler (Thermo Fisher). PCR master mix was prepared having a final volume of 25.0 µl for each reaction. This consists of 5.0 µl of genomic DNA, 2.5 µl of 10X PCR buffer, 0.75 µl of 50 mM MgCl_2_, 0.5 µl of 0.5 mM dNTP mixture, 1.0 µl of 10 pmole primer stock solution, 1.25 µl of DMSO, 13.8 µl of nuclease- free water and 0.2 µl of Taq DNA polymerase (5.0 units/µl) (Cat# 10342053, Invitrogen – Thermo Fisher Scientific). PCR reaction was performed that includes all DNA samples, one negative control reaction. The reaction mix was prepared for all samples and added into 200 µl PCR tubes. Genomic DNA was added later to each tube. Thermal cycling conditions were initiates at 95 °C, 30-second initial denaturation to get the DNA apart. It consists of 45 cycles of 95 °C, 10-second denaturing, 45 °C, 10-second annealing for binding of primers, and an extension of 60 °C, 30 s for synthesis of DNA. A final extension step at 60 °C for 5 min ensures that the amplification is thorough. The reaction is then stored at 4 °C until it is used again, keeping the amplified DNA ready for downstream uses. Agarose gel electrophoresis of the PCR products was performed using 2% (w/v) agarose gel using 0.5X TBE gel electrophoresis. Upon successful amplification, PCR product was purified by EXO-SAP IT PCR purification Kit (Thermo Fisher) and sequence using ABI Big Dye Terminator V3.1 cycle sequencing reaction kit (Applied Biosystems, USA). The sequencing data analysis was performed using V5.2 (Applied Biosystem, USA). Trimmed high quality data was used for identification up to species level using NCBI BLAST tool (Routtu et al. 2014). Phylogenetic analysis was performed using the neighbor-joining method in MEGA11, with a bootstrap test of 500 replicates. Neighbor- Joining (NJ) for 18 S rRNA trematodes is a widely used distance approached that reconstructs trees by minimizing total branch length, yielding both topology and branch lengths efficiently useful when the marker is conserved (like 18 S rDNA) and alignments are unambiguous (Saitou and Nei 1987). For small - medium datasets typical of species level surveys, NJ has demonstrated competitive accuracy while being computationally efficient (Tamura et al. 2004). It also remains as a common in marker - gene phylogenetics (including 18 S) where genetic distance (e.g., K2P) summarizes substitution patterns effectively (Wu et al. 2015). Meanwhile 500 bootstrap replicates is a nonparametric bootstrapping (resampling alignment columns with replacement) in which it can assess repeatability of clades (Felsenstein 1985). It is within the evidence based as its empirical and theoretical work shows that 100–500 replicates typically stabilize support values for many datasets; more may be needed only for especially difficult problems. Thus, 500 bootstrap replicates provide a pragmatic balance between precision and compute time (Pattengale et al. 2010).

## Results

**Fig. 2:**
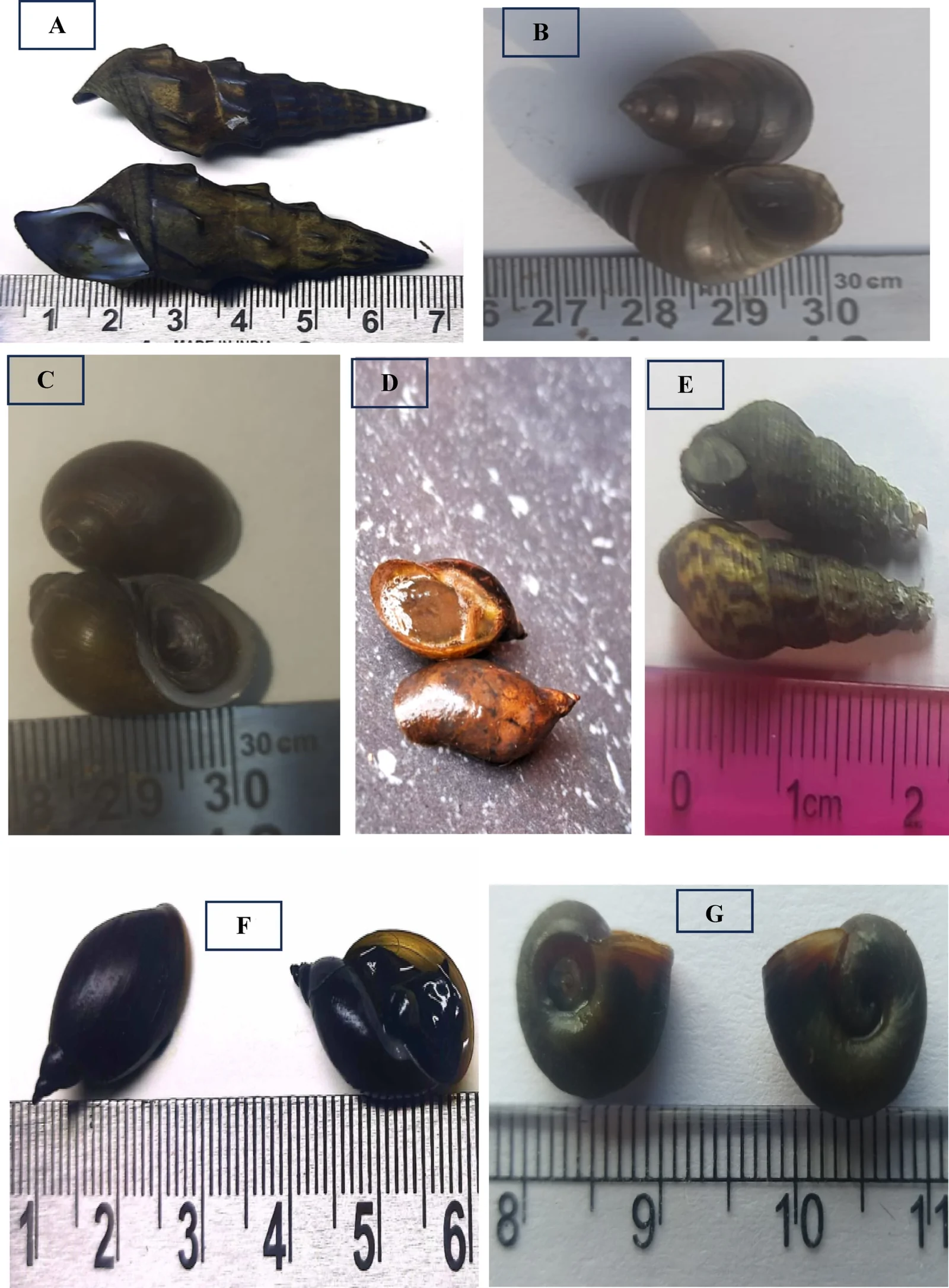
Morphological identification of freshwater snails- *Bortia costula* (**A**); *Filopaludina bengalensis* (**B**); *Paludomous pustulosa* (**C**); *Racesina luteola* (**D**); *Melanoid tuberculota* (**E**); *Radix rufescens* (**F**); *Indoplanorbis exustus* (**G**)

A total of 9467 freshwater snails were collected from three study areas in Manipur, India. They were sorted into seven types based on their physical features: *Bortia costula* (2403), *Filopaludina bengalensis* (1869), *Paludomus pustulosa* (1474), *Racesina luteola* (1147), *Melanoides tuberculata* (891), *Radix rufescens* (869), and *Indoplanorbis exustus* (814), as shown in Fig. 2. The overall trematode infection rate of snails is 0.26%. Table 1 presents the total number of snails examined, area-wise distribution of snail host species, and infection of trematode cercariae during the study period from January 2020 to April 2024.

**Table 1:** Area wise distribution of snail hosts species and infection of trematode cercariae during the study period from January 2020 to March 2024

Location	Snail sp.	No. of snails	No. of + ve snails (%)	Cercarial type isolated
Luwangsangbam matai	*B. costula*	1013	3(0.29)	Gymnocephalous cercariae, Lophocercous cercariae
	*F. bengalensis*	854	0	
	*I. exustus*	351	0	
	*P. pustulosa*	641	0	
	*R. luteola*	269	0	
Total	*=*	3128	3(0.09%)	
Motbung	*B. costula*	577	0	
	*F. bengalensis*	388	0	
	*I. exustus*	280	0	
	*P. pustulosa*	421	0	
	*M. tuberculota*	644	4(0.62)	Xiphidio cercariae
	*R. luteola*	669	4(0.59)	Echinostomous cercariae, Furcocercous cercariae
	*R. rufescens*	662	5(0.75)	Apharyngeate ocellate furcocercariae, Furcocercous cercariae
Total	*=*	3661	13(0.36%)	
Moreh	*B. costula*	813	4(0.49)	Gymnocephalous cercariae, Lophocercous cercariae
	*F. bengalensis*	627	1(0.15)	Gymnocephalous cercariae, Echinostomous cercariae
	*I. exustus*	183	0	
	*P. pustulosa*	412	0	
	*M. tuberculota*	227	4((1.76)	Xiphidio cercariae
	*R. luteola*	209	1(0.47)	Furcocercous cercariae
	*R. rufescens*	207	1(0.48)	Furcocercous cercariae, Apharyngeate ocellate furcocercariae
Total	*=*	2678	11(0.41)	

**Fig. 3:**
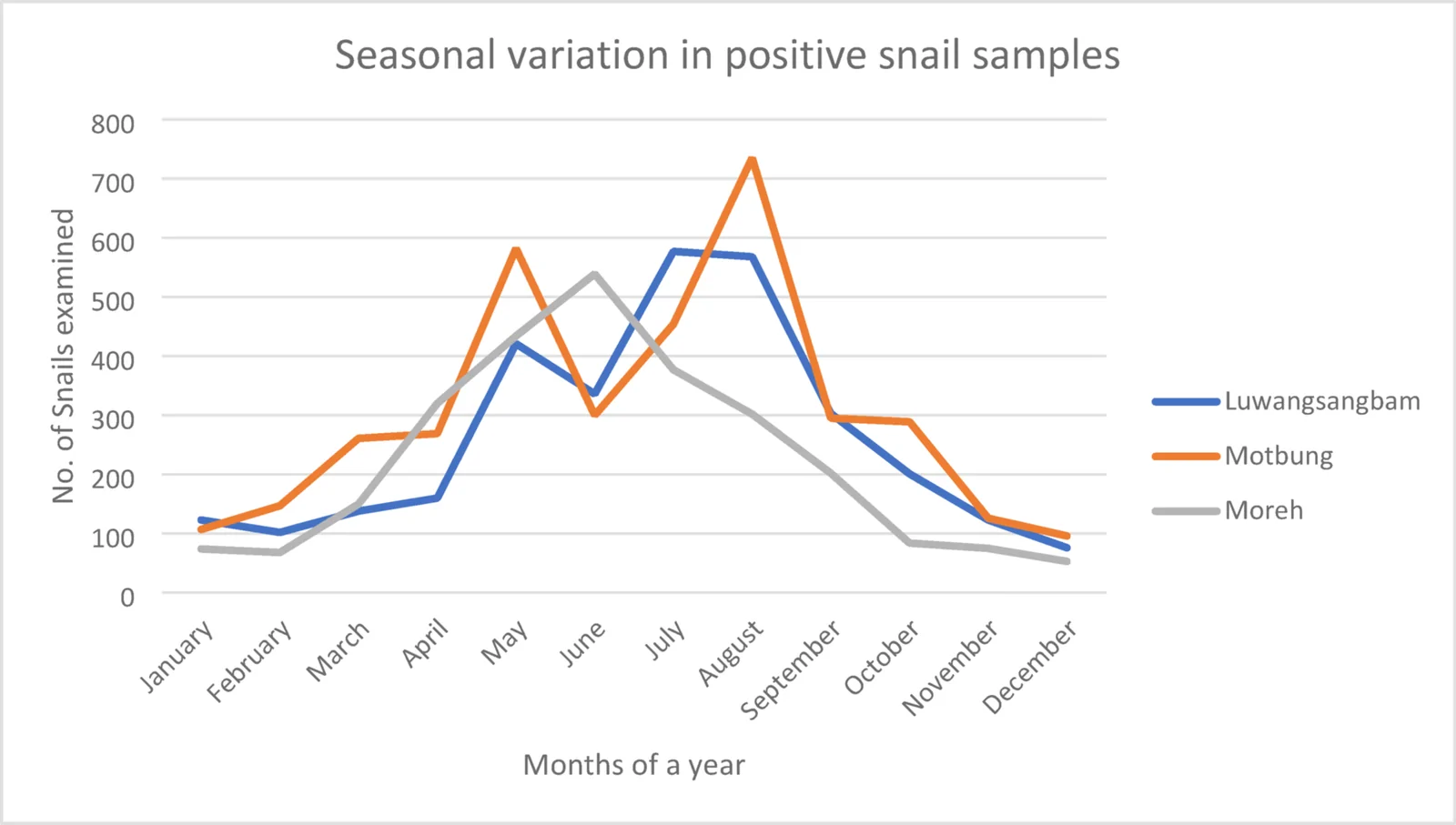
Month-wise distribution of trematode cerceriae positive snails during the study period

The highest cercariae infection rate of 0.41% was observed in the snails collected from Moreh, followed by 0.36% and 0.09% infection in the snails collected from Motbung and Luwangsangbam Matai, respectively. Table 2 presents the cumulative percentages of cercariae infection observed in the snail populations. Among the snail species, *M. tuberculota* revealed the highest cercariae infection (0.90%), followed by *R. rufescens* (0.69%), *R. luteola* (0.44%), *B. costula* (0.29%), and *F. bengalensis* (0.05%). Most of them revealed infections with multiple types of trematodes cercariae. On the contrary, *P. pustulosa* and *I. exustus* have not exhibited any trematode cercariae. The isolation rate of cercariae from the snail hosts was high during the period from late spring to summer and very low during winter and early spring showing, the seasonal variations of infection of trematode species in freshwater snails (Fig. 3). This information gives us important clues about the importance of temperature and rainfall in the life cycle trematodes species. It shows the prevalence of cercarial infection varies according to locations, across snail species and indicating a temperature-dependent parasite activity.

**Table 2:** Cumulative percentage of infected snails with cercaria during the study period from January 2020 to March 2024

Snail species	Number examined	No of + ve snails (%)	Cercarial types
*B. costula*	2403	7 (0.29%)	Lophocercous cercariae and Gymnocephalous cercariae
*F. bengalensis*	1869	1 (0.05%)	Gymnocephalous cercariae and Echinostomous cercariae
*I. exustus*	814	0	–
*P. pustulosa*	1474	0	–
*M. tuberculota*	891	8 (0.90%)	Xiphidio cercariae
*R. luteola*	1147	5 (0.44%)	Echinostomous cercariae Furcocercous cercariae
*R. rufescens*	869	6 (0.69%)	Furcocercous cercariae Apharyngeate ocellate furcocercariae
Total	= 9467	= 27 (0.26%)	

**Fig. 4:**
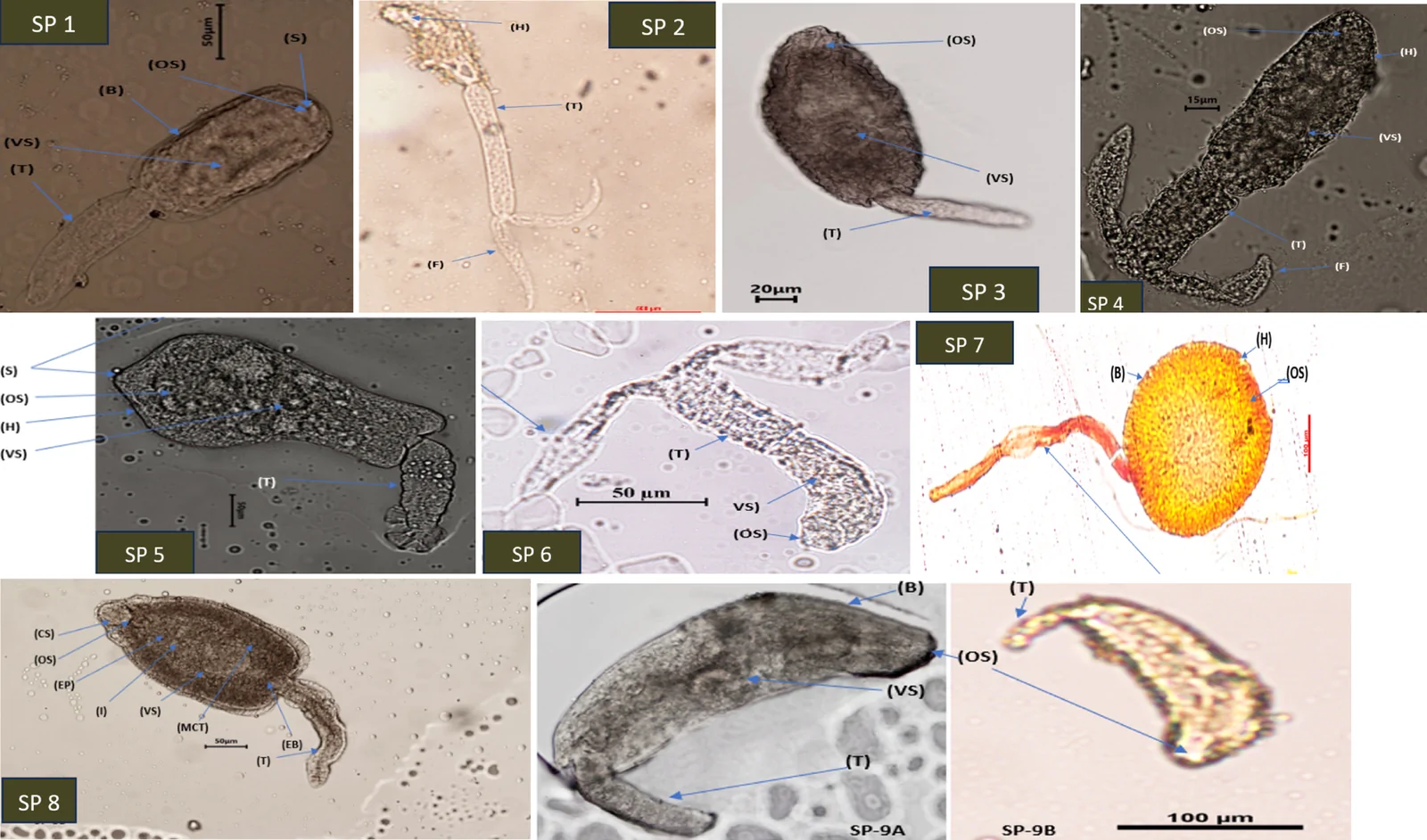
Microphotograph of cercariae (Stereo Zoom microscope: NIKON 200, Camera: NIKON Y- TV 55) Scale bar = µm. SP 1 Lophocercous cercariae, SP 2Apharyngeate ocellate furcocercariae, SP 3 *Gymnocephalous* cercariae, SP 4 Furcocercous cercariae, SP 5Xiphidio cercariae, SP 6 Furcocercous cercariae, SP 7 Gymnocephalous cercariae, SP-8 Echinostomous cercariae, SP-9 A (not to be scale) and 9BEchinostomous cercariae. H = Head organ, B = Body, T = Tail, F = Furca of tail, OS = Oral sucker, VS = Ventral sucker, S = Spine, S = Spine, OS = Oral sucker, CS = Collar spines, VS = Ventral sucker, T = Tail, F = Furca of tail, P = Pharynx, A = Acetabulum, EV = Excretory vesicle, I = Intestine, EP = Esophagus, MCT = Main collecting tube, EB = Excretory bladder

**Fig. 5:**
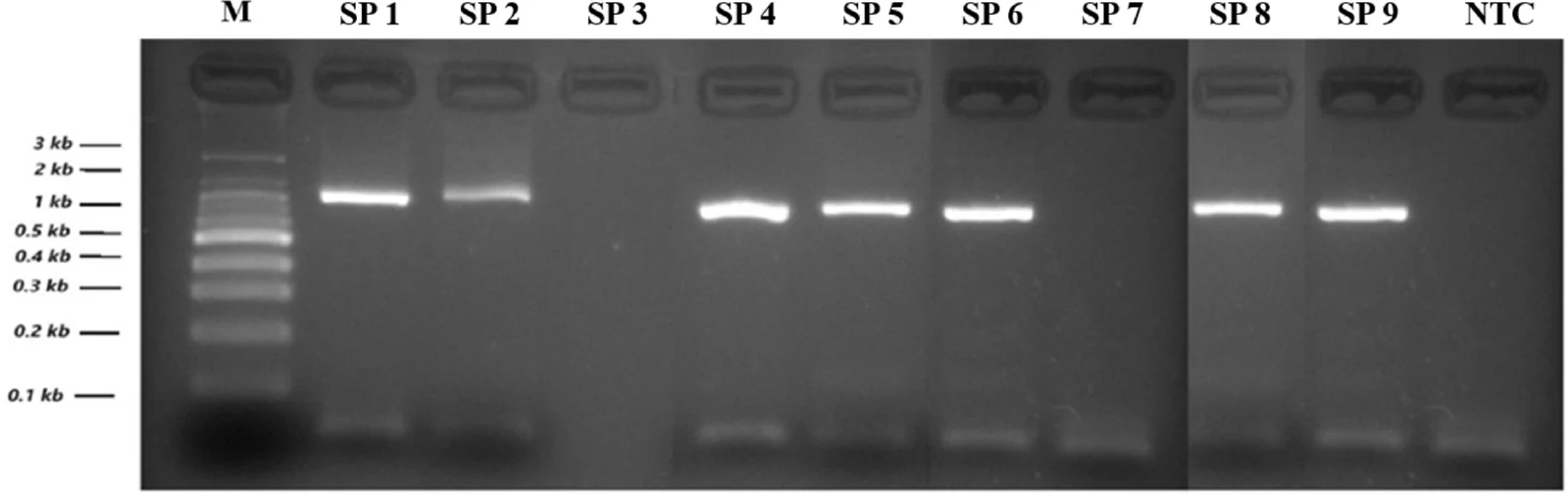
Agarose Gel electrophoresis of PCR products using 0.5X TBE electrophoresis buffer (the size of targeted gene is ~900 bp)

**Fig. 6:**
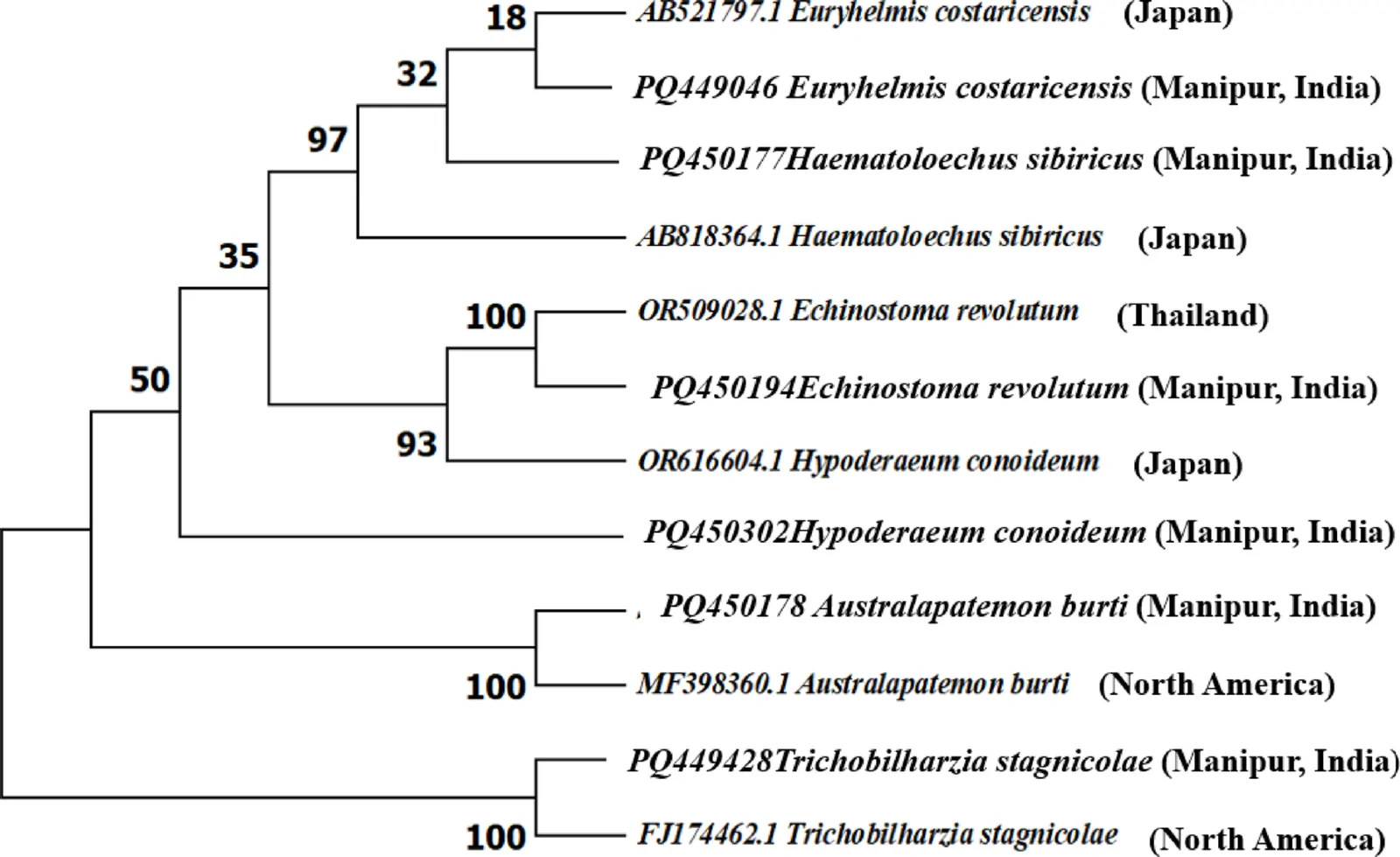
Phylogenetic analysis of the trematodes infected in different snails

The microscopy examination of the wet mount and carmine stained smears of the six different morphotypes of trematode larvae had revealed nine types of cercariae labelled as SP 1 = Lophocercous cercaria of *Euryhelmis* sp., SP 2 = Apharyngeate ocellate furcocercaria of *Trichobilharzia* sp., SP 3 = Gymnocephalous cercaria of *Fasciola* sp. SP 4 = Gymnocephalous cercariae of *Psilostomidae* sp., SP 5 = Furcocercous cercaria of *Australapatemon* sp., SP 6 = Xiphidio cercaria of *Haematoloechus* sp., SP 7 = Furcocercous cercariae of *Australapatemon* sp., SP 8 = Echinostomous cercaria of *Echinostoma* sp., SP 9 = Echinostomous cercariae of *Hypoderaeum* sp. (Fig. 4) for molecular analysis. Gel electrophoresis confirmed the presence of intact genomic DNA for most samples, except SP3 and SP7, which showed partial degradation. As the primers B forward and D reverse were used, the size of amplified product obtained was found to be near to 900 bp band on gel (Fig. 5). As per the primer BLAST results the size of band can be estimated as ~880 bp. The sequences (FASTA format) were generated from each sample and BLAST analysis give out the prospect of species identification of cercariae and, in turn validate species identification (Table 3). Using the Neighbor - Joining method in MEGA 11 to look at evolutionary relationships between single taxa and groups of taxa gives us a lot of information about how the trematode species we look at are genetically different and how they are related to each other (Fig. 6). High bootstrap values (from 500 replicates) confirm the reliability of the inferred phylogenetic relationships among trematode species.

**Table 3:** Results of BLAST analysis

Sl. nos.	Sample name	Cercarial type	Gene bank accession no	B- For Seq length(bp)	BLAST HIT	D-Rev Seq len (bp)	BLAST HIT
1	SP1	Lophocercous cercariae	PQ 449,046	530	*Euryhelmis costaricensis* (97%)	566	*Euryhelmis costaricensis* (97%)
2	SP2	Apharyngeate ocellate furcocercariae	PQ449428	481	*Trichobilharzia stagnicolae* (96%)	706	*Trichobilharzia stagnicolae*
3	SP3	Gymnocephalous cercariae	NA	–	–	–	–
4	SP4	Furcocercous cercariae	NA	253	*Australapatemon burti* (93%)	697	*Apatemon* sp (87%)
5	SP5	Xiphidio cercariae	PQ450177	394	*Haematoloechus sibiricus* (99%)	775	*Haematoloechus longiplexus* (97%)
6	SP6	Furcocercous cercariae	PQ450178	391	*Australapatemon burti* (99%)	690	*Apatemon* sp (99%)
7	SP7	Gymnocephalous cercariae	NA	–	–	–	–
8	SP8	Echinostomous cercariae	PQ450194	508	*Echinostoma revolutum* (99%)	829	*Echinostoma revolutum* (99%)
9	SP9	Echinostomous cercariae	PQ450302	402	*Hypodereum conidium* (96%)	616	*Hypodereum conidium* (85%)

## Discussion

Investigating the trematode populations within freshwater snail communities in specific ecosystems offers critical knowledge about trematode ecological dynamics. This investigation aimed to determine the distribution of snail intermediate hosts harboring diverse trematode taxa, including *Paragonimus* species, in Manipur, India. A comprehensive analysis by Selbach et al. (2020) examined 5,347 snails encompassing six species from German lakes, revealing infections with 36 distinct trematode species. According to Rachprakhon and Purivirojkul (2024), their 2018–2019 seasonal analysis revealed 1275 snails infected with 15 unique morphotypes, yielding a 1.8% infection prevalence.

The present study documented cercarial infections among 9,467 freshwater snails across three research locations in Manipur. Analysis revealed that 0.26% of freshwater snails harbored trematode cercariae of various species endemic in the state of Manipur. Multiple cercarial species were identified throughout the investigation period, spanning 2020 to 2024, in Manipur. This fluctuation in infection prevalence could be partially explained by the progressive reduction in viable habitats for potential intermediate and definitive hosts of specific digenean trematodes in this geographical region. Similar investigations conducted in Bangkok, Nakhon Nayok, and the Chao Phraya Basin have demonstrated cercarial infection rates of 4.3%, 4.7%, and 5.9%, respectively (Gál et al. 2019; Wiroonpan et al. 2021). Analogous observations emerged from two earlier studies in Thailand, where trematode infection rates of 0.20% and 4.7% in freshwater and brackish water snails, respectively, were documented by Chontananarth et al. (2017) and Pratumsrikajorn et al. (2017). Whereas Tigga et al. (2014) and Sharatkumar and Mohilal (2006) observed elevated prevalence rates of 7.33% in Ranchi and 16.94% in Manipur, respectively. Research in Sri Lanka by Jayawardena et al. (2011) revealed a 16% infection rate in freshwater snails sampled from three climatic zones. The reduced infection rates in wild snail populations may be attributed to substantial parasite-induced mortality and seasonal fluctuations in snail species populations.

Epidemiological analysis by Rachprakhon and Purivirojkul (2024) demonstrated elevated cercarial infection rate (5.9%) in precipitation periods, indicating meteorological parameters’ substantial influence on parasite-host relationships. This observation remained consistent across multiple cercarial morphological variants and aligned with the observation made by Dumidae et al. (2024) which established that climatological variable, including aquatic thermal conditions and precipitation patterns, fundamentally impact parasite-host ecological dynamics. This study revealed pronounced temporal fluctuations in trematode infection prevalence, with maximum infection rates occurring between May and September, suggesting significant correlations with environmental parameters including thermal conditions and atmospheric moisture. The main reasons for low infection during winter might be due to low chances of interactions between miracidium and snail hosts, migration of reservoir hosts, and decrease in snail population.

In the preceding investigation, *Bortia costula*, *Filopaludina bengalensis*, *Paludomous pustulosa*, and *Indoplanorbis exustus* were documented as natural susceptible primary intermediate snail hosts of trematodes prevalent in Manipur, demonstrating infection frequencies of 1.83%, 0.90%, 0.23% and 0%, respectively, during 2018–2019 (Yumnam et al. 2024). Significantly, the mollusc species *I. exustus* demonstrated no trematode infection in this analysis, which is in complete disagreement with the findings of Sharatkumar and Mohilal (2006) who documented a 22.3% infection rate in *I. exustus* in Manipur. In our examinations, snail species *M. tuberculota* demonstrated the highest infection rates of 0.90% with Xiphidio cercariae while snail species including *P. pustulosa*, *I. exustus* exhibited no infections. This emphasizes the importance of host specificity in trematode life cycles and transmission patterns. Despite the meticulous examination no *Paragonimus* cercariae was detected of all the snail samples. The main reason for not finding *Paragonimus* cercariae-positive snails might be due to the rapid urbanization and industrialization that resulted in the significant change in the ecology and reduction in the natural habitation of the animal reservoir hosts of *Paragonimus*. Also, human activities, such as hunting and killing the reservoir animal hosts of *Paragonimus* and mass-scale collection of the intermediate snail hosts for human consumption from the endemic areas, might have significantly reduced the transmission of the parasite miracidia to the snails.

## Conclusion

The current research provides a notable advancement in understanding the interactions between the freshwater snails and the trematodes prevalent in Manipur, India. The combination of morphological, molecular, and ecological methodologies has facilitated a thorough evaluation of trematode species, revealing insights into taxa and snail species that were previously undetermined. Six trematode species namely *Euryhelmis costaricensis*, *Trichobilharzia stagnicolae*, *Australapatemon burti*, *Haematoloechus sibiricus*, *Echinostoma revolutum*, and *Hypoderaeum conoideum* were characterized at morphological and molecular levels. The study revealed that the freshwater snails *B. costula*, *R. luteola. M. tuberculota* and *R. rufescens* are naturally occurring permissive hosts of multiple trematode cercariae, whereas *I. exustus* and *P. pustulosa* are non or less permissive to trematode infection. A thorough study in more local areas, especially in the remote region of Manipur, is needed to find the first intermediate snail host of *Paragonimus* and other trematode species. The research outcomes in this study, showed substantial relevance for understanding trematode distribution and infection patterns within Manipur’s geographical boundaries. The detected minimal prevalence rates indicate that trematode parasitism potentially poses limited risk to gastropod populations and public health concerns in the region. However, the identified geographic and seasonal fluctuations in infection frequencies suggest heightened transmission risks during specific periods and locations. The differential susceptibility among gastropod species to trematode infections emphasizes the importance of species-specific monitoring and intervention protocols.
